# Barriers and facilitators to reducing low-value care for the management of low back pain in Iran: a qualitative multi-professional study

**DOI:** 10.1186/s12889-023-17597-1

**Published:** 2024-01-17

**Authors:** Seyedeh Yasamin Parvar, Parviz Mojgani, Kamran Bagheri Lankarani, Fereshteh Poursaeed, Leila Sadat Mohamadi Jahromi, Vinaytosh Mishra, Alireza Abbasi, Saeed Shahabi

**Affiliations:** 1 Health Policy Research Center, Institute of Health, Shiraz University of Medical Sciences, Shiraz, Iran; 2 Student Research Committee, Shiraz University of Medical Sciences, Shiraz, Iran; 3 Iran-Helal Institute of Applied Science and Technology, Tehran, Iran; 4 Research Center for Emergency and Disaster Resilience, Red Crescent Society of The Islamic Republic of Iran, Tehran, Iran; 5 Transitional Doctor of Physical Therapy Program, College of Professional Studies, Northeastern University, Boston, USA; 6 Department of Physical Medicine and Rehabilitation, School of Medicine, Shiraz University of Medical Sciences, Shiraz, Iran; 7 College of Healthcare Management and Economics, Gulf Medical University, Ajman, UAE

**Keywords:** Low-value care, Low back pain, Rehabilitation, Choosing wisely, Health policy, Qualitative study, Iran

## Abstract

**Introduction:**

Low back pain (LBP) is a prevalent musculoskeletal disorder with a wide range of etiologies, ranging from self-limiting conditions to life-threatening diseases. Various modalities are available for the diagnosis and management of patients with LBP. However, many of these health services, known as low-value care (LVC), are unnecessary and impose undue financial costs on patients and health systems. The present study aimed to explore the perceptions of service providers regarding the facilitators and barriers to reducing LVC in the management of LBP in Iran.

**Methods:**

This qualitative descriptive study interviewed a total of 20 participants, including neurosurgeons, physiatrists, orthopedists, and physiotherapists, who were selected through purposive and snowball sampling strategies. The collected data were analyzed using the thematic content analysis approach.

**Results:**

Thirty-nine sub-themes, with 183 citations, were identified as barriers, and 31 sub-themes, with 120 citations, were defined as facilitators. Facilitators and barriers to reducing LVC for LBP, according to the interviewees, were categorized into five themes, including: (1) individual provider characteristics; (2) individual patient characteristics; (3) social context; (4) organizational context; and (5) economic and political context. The ten most commonly cited barriers included unrealistic tariffs, provider-induced demand, patient distrust, insufficient time allocation, a lack of insurance coverage, a lack of a comprehensive referral system, a lack of teamwork, cultural challenges, a lack of awareness, and defensive medicine. Barriers such as adherence to clinical guidelines, improving the referral system, improving the cultural status of patients, and facilitators such as strengthening teamwork, developing an appropriate provider-patient relationship, improving the cultural status of the public, motivating the patients, considering an individualized approach, establishing a desirable payment mechanism, and raising the medical tariffs were most repeatedly stated by participants.

**Conclusion:**

This study has pointed out a great number of barriers and facilitators that shape the provision of LVC in the management of LBP in Iran. Therefore, it is essential for relevant stakeholders to consider these findings in order to de-implement LVC interventions in the process of LBP management.

**Supplementary Information:**

The online version contains supplementary material available at 10.1186/s12889-023-17597-1.

## Introduction

Healthcare is frequently categorized as “high-value” or “low-value” procedures [1]. High-value care (HVC) is described as the best treatment for the patient, producing the optimal outcome given the conditions, and being provided at the appropriate cost [2]. HVC promotion initiatives are becoming more widespread in medicine, mainly via educational curricula and guidelines [3, 4]. On the other hand, low-value care (LVC) is defined as a service that provides no benefit in particular clinical situations [5].

Negative consequences of LVC can be seen in low-value procedures in hospitals [6], testing in low-risk patients [7], and high rates of unnecessary specialist consultations [8], as these may not have demonstrable benefits to patients or their risks exceed projected advantages. Although various issues are involved in the LVC administration, physicians’ concerns about probable negative impacts on patients’ healthcare experiences are frequently highlighted as barriers to de-implementation [9–12]. Researchers have identified provider-related factors that significantly influence the continuation of LVC. For instance, a significant portion of a healthcare provider’s daily practice is habitual rather than intentional, as routines don’t require conscious decision-making [13, 14]. Finding the factors contributing to LVC in a practice environment, creating multimodal curricula to address skill and knowledge gaps, and changing the medical institution’s culture can help reduce LVC over time [15].

De-implementation is becoming increasingly important in efforts to improve the quality, cost, and fairness of healthcare [16, 17]. Of note, in developing countries like Iran, LVC is responsible for millions of dollars being spent on unnecessary healthcare, and there are also inequalities in the provision of healthcare services that benefit the wealthy [18–20]. One strategy that the health system may use to control costs is to limit patient choices to preselected high-value options. But this method might not take into account what the patient wants when it comes to important parts of care [21, 22].

Low back pain (LBP) is the most prevalent musculoskeletal condition [23] and remains the leading cause of years lived with disability (YLDs) on a global scale [24]. The 2018 Lancet Low Back Pain Series brought to light the rising global burden of LBP, which is partly caused by poor medical care [25–27]. A wide array of management and treatment options, ranging from conservative therapies to complex surgical procedures, may be considered based on the patient’s condition. In primary care settings, most patients with acute LBP are sent for radiology-based diagnoses that are unnecessary, may be harmful, and are expensive for both the patient and the healthcare system [28]. Multiple national guidelines for the diagnosis and treatment of LBP have been published to provide related evidence-based recommendations. The use of narcotics, referrals to other doctors, and advanced diagnostic imaging are used more often. However, physiotherapy remains the most preferred choice among physicians [29].

Implementing systemic reforms in the healthcare system may be needed to enhance the likelihood of aligning interventions for LBP with established guidelines. In the absence of red flags, such as a history of major trauma, pain lasting more than six weeks, being younger than 18 or older than 50 years, having constitutional symptoms, improving direct access to non-prescriptive health care providers, and increasing timely referrals from primary care and specialist healthcare providers, this would increase the proportion of people who receive early non-pharmacological treatment [30]. Therefore, improvements in LBP management represent a possible area of cost savings for the healthcare system while simultaneously increasing the quality of service as a result of the skyrocketing cost of healthcare [29].

The extent of unnecessary usage of medical services, especially in the management of LBP, is significant in Iran’s healthcare system [31]. Usually, many patients with LBP refer to not only general practitioner (GP) but also orthopedists, rheumatologists, and physiatrists seeking X-rays for further diagnostic evaluation and unnecessary drugs without considering the efficacy of physiotherapy and other home-based rehabilitations [32]. Unfortunately, there are no organized and step-by-step guidelines for using imaging assessments, utilizing narcotics, and referring to other physicians based on their specialty in the management of LBP in Iran. Since the utilization of LVC for LBP appears to be high in Iran, we designed this study to explore the experiences of relevant healthcare professionals regarding common barriers and facilitators to reducing LVC in managing LBP.

## Methods

This qualitative interview-based descriptive study was conducted from April 2022 to March 2023 in Fars, Iran. To improve the methodological and reporting quality, both the “Critical Appraisal Skills Program (CASP) Qualitative Checklist” [33] and “Standards for Reporting Qualitative Research (SRQR)” [34] were considered during the conceptualizing, designing, conducting, and reporting the results. The Institutional Review board of Shiraz University of Medical Sciences previously assessed and approved the study proposal (No. 26,909).

### Participants

Both purposive and snowball sampling approaches were used to recruit the participants. In this regard, a list of potential participants, including orthopedic surgeons, physiotherapists, physical medicine and rehabilitation specialists, and rheumatologists practicing in Fars Province, was prepared using the Islamic Republic of Iran Medical Council and Iranian Scientometrics Information Database websites. To achieve the highest attainable diversity, the research team attempted to select practitioners and faculty members with varying characteristics in terms of gender, occupation, clinical experience, and specialty. After each interview, the interviewer was asked to introduce another individual who could provide valuable information on the subject of the research. Participant selection and interviews continued until data saturation was achieved and no new information could be obtained. The last five interviews with repetitive data were considered to ensure data saturation. To coordinate the interview session, a consent form containing general project information and ensuring adherence to ethics was sent to the participant through email or an instant messaging app. In this form, participants were guaranteed that their identities would remain anonymous throughout the study and that the files would be eliminated after the study was completed. The interview was scheduled after the participant had consented to attend the interview.

### Data collection

Semi-structured interviews were conducted in both online and in-person formats by the first author, a final-year female medical student well-qualified in qualitative research and semi-structured interviews. Furthermore, as a team, all the authors recognized the importance of ensuring the quality of the interviews. Before commencing the project, we held several preparatory meetings to practice experimental interviews. A comprehensive explanation of the study objectives was given to the participants a few days before the interview. An interview guide containing open-ended questions was used to facilitate the management of the interview flow (Table 1). The interview questions were evaluated during initial interviews and revised based on feedback from the interviewees to improve clarity. Interview sessions were recorded by a sound recorder from the beginning of the interviewer’s speech to its end, excluding the introduction and appreciation. The interviewer also took notes during interviews to facilitate data analysis. After each interview was conducted, the recorded file was transcribed, and after adding the notes, it was saved in Word Office software anonymously.

**Table 1:** Interview guide including open-ended question and relevant probes

• **Experiences**	
• What are your perspectives and reflections on the definition of low-value care? Can you provide some examples of examinations in management of low-back pain that will fall under this definition?	
• Can you explain to me your opinions about low-value care?	
• What do you think are the most important reasons for prescribing low-value care in the management of LBP?	
• How do you think of the following effects on the use of low-value care in the management of LBP?	
• Economics	
• Organizational structures	
• Financial factors	
• Payment factors	
• Governance factors	
• Socio-economic factors	
• Environmental mechanisms	
• Individuals (patients, providers, etc.)	
• Have you considered any approach to curb the use of low-value care in the management LBP? If so, what did you do, and how did the adopted strategy work?	
• **Probable future measures**	
• In your perspective, what are the practical strategies to diminish the use of low-value care in the management of LBP in Iran?	
• What can make adopted strategies unsuccessful?	
• **Final recommendations**	
Is there anything else you think it is important for us to know?	

### Data analysis

The data analysis process was carried out in parallel with the data collection, and the thematic content analysis approach was applied. The coding of the collected data was iteratively performed by two authors (SYP and FP). Then, four authors (SYP, SSH, AA, and LSMJ) independently compared, argued, and evaluated the identified codes and sub-themes, unraveling the final themes. The results of the data analysis were provided to participants for confirmation. In fact, to ensure greater consistency and guarantee an appropriate interpretation, methodological triangulation was used, involving interviewers, co-authors, and participants in the analysis of the data. This process was critically monitored and appraised by the expert author (KBL). Any discrepancies among the authors in the data analysis process were resolved through discussion sessions. This step was conducted manually.

### Rigor and trustworthiness

Several methods have been considered to certify the rigor and trustworthiness of qualitative studies by enhancing the confirmability, credibility, dependability, authenticity, and transferability of findings [35]. To cover such concepts, the research team adopted several strategies, including: (1) reviewing and confirming the results of analysis by participants (confirmability); (2) prolonged engagement of the first and corresponding authors throughout the projects and checking the findings by relevant experts (credibility); (3) involving several authors with different executive and scientific experiences in data analysis (dependability); (4) considering quotes from almost all participants throughout the manuscript (authenticity); and (5) recruiting samples with different specialties and clinical experiences (transferability).

## Results

Twenty-two participants, including six physiatrists, five physiotherapists, five rheumatologists, and six spine surgeons (three orthopedists and three neurosurgeons), were contacted. After transcribing and coding 15 interviews, we felt that we had reached saturation. To test our theory, we interviewed five more people. Since no information was added following these interviews, we did not pursue additional participants. Therefore, the team did not feel it was necessary to conduct any additional interviews. Due to privacy concerns and the low quality of the recorded interview, we did not include a female rheumatologist and a male physiotherapist in the study. In total, we interviewed 20 participants, including 11 females and nine males, through a combination of seven in-person and 13 virtual interviews, with a mean duration of 25.6 min (SD 12.2). The demographic characteristics of the participants are represented in Table 2.

**Table 2:** Demographic characteristics of participants

ID	Sex	Age (years)	Clinical experience (years)	Specialty	Interview format	Interview duration
**01**	M	46	28	Physiotherapy	Virtual	50.35
**02**	F	35	8	Physical Medicine & Rehabilitation	Virtual	17.28
**03**	M	30	4	Orthopedics	Virtual	8.22
**04**	F	57	33	Rheumatology	In-person	33.05
**05**	F	33	11	Physical Medicine & Rehabilitation	Virtual	32.55
**06**	M	27	5	Orthopedics	Virtual	22.58
**07**	M	43	18	Physical Medicine & Rehabilitation	Virtual	49.43
**08**	M	35	10	Neurosurgery	Virtual	17.57
**09**	F	33	10	Rheumatology	In-person	17.37
**10**	M	36	13	Neurosurgery	In-person	37.08
**11**	F	48	19	Physical Medicine & Rehabilitation	In-person	22.27
**12**	F	28	6	Physical Medicine & Rehabilitation	In-person	13.37
**13**	F	40	16	Physical Medicine & Rehabilitation	In-person	32.12
**14**	F	40	15	Physiotherapy	Virtual	44.46
**15**	F	32	10	Physiotherapy	Virtual	18.55
**16**	F	33	10	Rheumatology	In-person	13.50
**17**	M	33	9	Neurosurgery	Virtual	19.25
**18**	F	36	11	Physiotherapy	Virtual	15.10
**19**	F	49	22	Rheumatology	Virtual	13.15
**20**	M	34	6	Orthopedics	Virtual	22.39

The following explains the barriers and facilitators in detail, and quotes from the in-depth interviews will be used for further clarity (Tables 3 and 4).

**Table 3:** Main barriers in reducing low-value care in the management of low back pain in Iran

Main themes	Sub-themes	Quotes	Participant ID
**Individual provider characteristics**	Non-adherence to clinical guidelines	*“Unfortunately, in our country, neither doctors, physiotherapists, nor our personnel adhere to the guidelines.” (1)* *“I do not request MRI until I have done a thorough physical examination. But for some neurosurgeons, the receptionist will not make an appointment if the patient doesn’t have an MRI. For example, if the patient has symptoms of limping or a listhesis the patient should visit a neurosurgeon, but they do not conclude until the patient has undergone an MRI.” (19)*	1, 11, 19
	Lack of trust among involved professionals	*“They may not know whether the physiotherapist is an expert in their work to refer the patient to them, and this concern makes them carry out the treatment alone.” (14)*	14
	Self-centered actions	*“A feeling of ownership towards the patient is formed in the specialists, which prevents them from referring patients to other specialists to receive high-value care treatment.” (2)* *“One thing that happens in our specialties is tunnel vision. Depending on their areas of expertise and the cases they typically visit, each doctor has a unique perspective on a patient. Low back pain may be more inflammatory for rheumatologists, or the mechanical aspect may be important for us, and we visit these patients more frequently.” (5)*	2, 5, 6, 7, 8, 14, 17
	Defensive medicine	*“When a patient comes to me, I cannot refer them to another healthcare professional without providing any medical service due to the fear of missed diagnoses, which can lead to overtreatment.” (5)* *“The extensive use of MRI and other paraclinical services has replaced the physical examination, and physicians don’t have enough patience for performing the examinations.” (1)*	1, 5, 6, 14, 16,17, 18,19
	Unawareness of providers	*“Despite the improvement of physiotherapy, there is no proper knowledge among physicians about it. Many of our medical practitioners’ knowledge about physiotherapy is in the form of articles, and because the doctor’s tool is surgery, they suggest surgery.” (14)* *“Lack of awareness, either from patients or service providers, is an important factor in using low-value care practices.” (18)*	13, 14, 15, 18
	Undesirable training	*“The expansion of medical courses and high-level education makes general practitioners not remember simple issues such as back pain red flags and lead patients to low-value care practices.” (5)* *“There is debate on specialists’ knowledge and experience.” (6)*	5, 6, 11, 13,16,17
	Inappropriate provider-patient communication	*“Many doctors prescribe the medical services requested by patients to satisfy the patients and not cause any issues from them.” (3)*	3, 7
	Lack of comprehensive examination	*“When a patient presents with low back pain, the first helpful approach that we should perform is a thorough physical examination. For example, if we perform the SLR and reverse SLR tests, we can to some extent understand whether LBP is associated with discopathy or radiculopathy. Based on the history and examination, some patients may have inflammatory spondyloarthropathies. We can order lab tests initially, and then if the pain doesn’t improve with conservative management, imaging and other costly services may be required.” (9)*	1, 7, 9, 11, 13, 16,17
	Not allocating enough time	*“I do not have that much time to explain and teach every patient. I see seventy patients in the clinic; If I want to spend five minutes explaining to each patient, I would spend six hours explaining to patients, and I don’t have this time in the clinic.” (10)* *“Perhaps due to the out-of-proportion number of patients, many of my colleagues have to visit patients and work beyond their capacities. And this causes the quality of their visits to decrease, and they cannot spend the appropriate amount of time in the clinic.” (13)*	5, 9, 10, 12, 13, 14, 17, 16,20
	No specific treatment for each patient	*“Treatment is done based on the approach of a certain range of patients.” (7)* *“In our field - physiotherapy - magnet therapy, laser therapy, or shock wave therapy, which are expensive treatments, can be effective if there is an indication, but sometimes doctors request these treatments for all patients.” (15)* *“Some doctors are not familiar with some physiotherapy equipment and request the same prescription for all patients.” (15)*	7, 15,18
	Provider induced demand	*“The cost of corticosteroid injections is much higher than physiotherapy treatments. So, doctors prefer to treat their patients with a single injection session rather than long-term conservative treatments.” (1)* *“Doctors, who visit low-back pain patients mostly, prescribe MRIs, not because of illiteracy or a lack of knowledge. It’s for finding an indication for surgery in an MRI to the extent that they can defend themselves in court.” (6)* *“The use of corticosteroid injections has increased, especially in the rehabilitation setting. These injections are mostly done in private centers for financial motives.” (20)* *“Attracting patients and also financial motives make a number of doctors prescribe expensive medical services without indication.” (15)*	1, 5, 6, 8, 10, 11, 12, 15, 18, 20
**Individual patient characteristics**	Lack of awareness	*“A patient who refers with low back pain complaints does not have much information and relies on questioning people around them to make their decisions.” (1)* *“The main issue with the high use of LVC services is that patients do not have knowledge about rehabilitation and its services, and at governmental clinics with low visit costs, they can access other specialists easily as well.” (5)* *“The level of knowledge our people have about medicine is very low.” (13)*	1, 5, 7, 13, 14, 15, 18, 20
	Distrust	*“Since the field of rehabilitation and the services that it provides in low back pain management are unknown to most people, and on the contrary, in terms of neurosurgery, we have renowned surgeons, the patient accepts a suggestion from someone who has credibility, and when we explain to the patient that surgery is not necessary, it is not acceptable to them.” (2)* *“Medical service requests are made by the patients since many patients do not trust their doctors.” (3)* *“Social media and misguided advertisements have reduced patient and doctor trust.” (9)* *“Imaging is prescribed at the insistence of the patients, and if we do not refer them for imaging, they will visit another doctor, or it may even be dangerous for the doctor’s life in small communities.” (9)*	2, 3, 7, 9, 10, 12, 14, 15, 17, 20
	Cultural challenges	*“In the sports medicine center where we worked, doing exercises was not culturally accepted as a treatment, and medications and injections were accepted as treatments.” (1)* *“Sometimes a one-day rest with a short-term painkiller is enough to treat the patients, but the patients do not accept it and expect us to do something incredibly special for them. They want to get secondary psychological care.” (2)* *“Cultural issues in our country lead patients to request excessive demand for a series of medical services.” (8)* *“When I was a GP, I tried to make patients aware, but I realized that when someone explained to the patient that these treatments are not effective and the condition would improve itself, to them, the patients would not accept this from us.” (2)* *“Many of these mismanagements go back to cultural factors; otherwise, the doctors know the indications at the right time, and if symptoms are present, they prescribe an MRI on time, and if there is an indication, they encourage the patients to undergo surgery.” (20)*	1, 2, 3, 4, 8, 9, 11,16, 20
	Demanding non-invasive interventions	*“Patients’ desire for minimally invasive procedures without anesthesia, without incisions, even though without any clear indication.” (8)*	8
	Non-adherence to prescribed interventions.	*“Sometimes, patients visit several medical practitioners to get the desired prescription, which can lead to subsequent practitioners making mistakes.” (11)*	1, 2, 9, 11, 12, 15
	Inappropriate cooperation of patients with providers	*“Due to the low socio-economic status of some patients, they do not follow the guidelines, and this causes non-cooperation.” (6)* *“Patients do not cooperate with medical practitioners.” (11)*	3, 6, 10, 11, 14
	Willingness to get better quickly	*“Many patients do not have patience and prefer to take multiple treatments or receive a treatment that provides quicker results.” (2)* *“Most of the time, a patient who needs non-surgical and long-term treatment—the kind of treatment that requires their cooperation—does not cooperate with the doctor and prefers to jump to the last treatment line very quickly. And they think if they undergo surgery, they will be relieved forever.” (11)* *“Mostly, patients are just looking for someone who tells them that their condition is not serious; otherwise, they do not seek treatment and do not follow up on it, and they end up getting the treatment. If we suggest the patient continue treatment by manual therapy and exercise, they will not continue the treatment.” (14)*	2, 3, 8, 11, 14,16, 18
	Demand for receiving technology-based interventions	*“A patient who cannot afford the cost of treatment will do an MRI only because of the psychological aspect, even if they need to borrow money. For example, I have visited an 80-year-old patient with diabetes, hypertension, and advanced arthritis of the spine who wants to do an MRI only for psychological aspects. And this will put his mind at ease that he has done an MRI even if he never undergoes an operation.” (20)* *“The more physiotherapy devices are used and the longer they are used, the more satisfied patients become.” (15)*	15, 20
**Social context**	Lack of team working	*“In our country, team working has not been accepted.” (1)* *“At the Ph.D. level in physiotherapy, we are trained to use ultrasonography for patient follow-up, and we should use it. But my experience shows that the radiology team, because of the experiences that they had, contravenes. But in our country, each specialty’s role is not clear.” (1)* *“Many surgeons prefer not to refer their patients to other specialists.” (2)* *“Teamwork is very weak in our country. It is mainly because, with the little knowledge that we gain, we tend to believe that we are experts in the field and can manage it ourselves, which prevents teamwork. We tend to comment on everything, and these interferences are largely a part of our sociology.” (13)*	1, 2, 3, 5, 7, 12, 13, 14, 18
	Inappropriate professional development	*“The same textbooks given to residents are advised to general medical students, which can confuse them.” (5)* *“Medical education at many universities does not include the rehabilitation department. So many GPs don’t know enough about rehabilitation. Therefore, in the surrounding villages, a patient with low back pain first refer to a GP, who then refers them without indication to a surgeon.” (5)* *“An intern spends only fifteen days in the neurosurgery ward with a heavy workload, and quality training is not possible during this limited timeframe.” (17)*	5, 13, 17
	Lobby by some high-power professional networks	*“A group of lobbying specialty has power on the level of the Ministry of Health to establish especial laws.” (10)*	10
	Low socioeconomic status of patients	*“In recent years, when scheduling an appointment to see me has become more difficult, it is uncommon to see a patient with low back pain as the first line of treatment. As a result, the patient who come to me have already invested money and time in imaging and have made significant effort to visit other specialties. Because these patients are typically of low socioeconomic status, they expect both diagnosis and treatment to be completed during their initial visit.” (7)*	7, 10, 17
**Organizational context**	Inadequate rehabilitation centers	*“The number of centers equipped with physiotherapists and sports medicine specialists in Iran is small.” (1)* *“Not everything is available everywhere. When we want to complete the examination of a patient, it may be necessary to prescribe EMG and NCV, and the patient should return to us the same day after that. But it may take a month to do it. So, we only prescribe X-rays that can be done quickly.” (17)*	1, 5, 6, 8, 17
	Availability of too many imaging centers	*“At the center where we work, MRI is at our fingertips, which increases the imaging request.” (4)* *“It is good that we have everything available, but it causes a waste of our resources. And this overtreatment of our patients is due to low prices, excessive availability of doctors, and faulty referral system.” (13)*	4, 7, 12, 13
	Lack of comprehensive referral system	*“Due to the crowdedness of the clinics or the lack of time spent by the medical practitioners on the patient’s physical examination, the patients are referred to do paraclinical exams in order not to miss anything.” (5)* *“In Iran, at the minimum time and the furthest point, medical services are accessible to patients.” (8)* *“Most of these are due to our faulty system, not our behavior. It means that I may want to behave properly and have enough knowledge and interest to do so. But when I enter this vicious economic, social, and cultural cycle, sometimes I have to do the third step first.” (7)* *“Patients make appointments with two or three rheumatologists at the same time without any restrictions, saying that they want to know the opinions of other rheumatologists.” (4)* *“The referral system is not appropriate. Many patients with low back pain can visit surgeons or other specialists as the first step in their treatment process. So, at the first level, the patient should visit a general practitioner, and they will be referred to a surgeon if they have red flags, or if physiotherapy intervention is needed, they should be referred to a physiotherapist.” (12)*	4, 5, 7, 8, 9, 10, 12, 13, 14
	Lack of insurance coverage	*“Exercise therapy is a significant part of low back pain management, but there is no defined tariff for it in our health system.” (1)* *“In Europe, acupuncture is covered by insurance free of charge for two phases of ten sessions a year, which is highly effective in reducing spasms, strengthening paravertebral muscles, and strengthening proprioception. So, for low-back pain, knee arthrosis, and migraine, they have entered their treatment system, and they need to pay less compared to surgery costs. While in our country’s government system, insurance has much better coverage for surgeries than manual therapy.” (11)* *“Insurance companies have a restrictive system, and we are disappointed with their changes and progress. In government sectors, insurance covers most of the cost, but, for example, it does not cover the cost of laser therapy, shock wave therapy, massage, or exercise. For example, we do massage, mobilization, teaching exercise, using physiotherapy equipment, and all the other necessary treatments because physiotherapist wants their patients’ conditions to improve. They do all these things; that is, we never say that because we did a twenty-minute massage, we will charge the patients this cost. I give massages to treat my patients, even for half an hour, and none of this is covered by insurance.” (14)* *“Unfortunately, because of the high number of patients in the public sector and the excessive costs in the private sector, for example, a rural patient who has a low income and cannot afford high medical costs but has canal stenosis and needs surgery can be mismanaged. Or a patient whose condition may improve with physiotherapy may round off their treatment with an injection.” (20)* *“Unfortunately, insurance coverage for the treatment of low back pain is not favorable for either the therapist or the patient because a limited maximum of treatment cost coverage for the patients are offered. For example, there may be patients who need ten or more physiotherapy sessions per season, and the insurance companies do not cover it unless they have specific insurance plans.” (18)*	1, 5, 7, 10, 11, 14, 15, 18, 20
	Undesirable payment mechanism	*“The patients visit doctors with voluminous medical records. It takes 45 min of our time evaluate them with thorough physical examination, but we receive the same amount of payment as other specialists get in a 15 min visit.” (4)* *“If we want to follow the guidelines, due to the special insurance system, teamwork will not be formed, and even if it is formed since everyone’s positions and roles are not defined, it may be disrupted.” (1)*	1, 4,20
	Lack of outcome-based payment	*“I think the main problem is time, and then for us, physiotherapists, or surgeons, there is no fee to be charged for these high-value care services to spend time on and teach exercises and correct patients’ lifestyle.” (5)* *“For example, I examine the patient for twenty minutes, and then it takes ten minutes to teach exercises and correct the lifestyle. No one pays me for the time spent, and very few doctors and other healthcare professionals are burdened with it.” (9)*	5, 9
	Delay in payments	*“Many doctors who have a reasonable number of patients prefer not to deal with insurance companies because they pay poorly. (2)* *“It is somewhat difficult to cooperate with insurance companies as they do not cover many services, and the deposits are delayed by six months to a year.” (15)*	2, 15
	Conflict of interests	*“There is a conflict between different fields, and they do not refer their patients to other specialists.” (12)* *“There are mediators who determine the treatment path for patients without understanding the guidelines and medical information about diseases; That is, from the beginning, when the patients get off the bus or plane, according to a process that has more of an economic perspective in it, more benefits go to the mediators than to the health system, doctors, laboratories, and pharmacies.” (7)* *“A series of interventions and medicines that we cannot use were previously said to be highly documented and recommended which in turn showed that they had economical conflict of interests.” (8)* *“The mafia power that exists in the food and drug industries is under the control of those who import minimally invasive surgery equipment’s, and they try to increase the acceptance of minimally invasive surgery among patients and doctors.” (8)*	7, 8, 10, 11, 12
	Lack of effective information system	*“Laboratory tests are recorded and saved in the database only for a month. Therefore, if I want to evaluate my patients’ conditions for the next four months, they have to visit me or other specialists again to get a prescription.” (9)*	9
**Economic and political context**	Un-realistic tariffs	*“Doctor’s fees and medical services are at low costs.” (4)* *“If my fee as a specialist is so high that patients can only visit me for special issues, then the referral system will be fixed.” (4)* *“In the field of rehabilitation, there is no specific tariff for exercise therapy, which has a high level of evidence in the treatment of low back pain. There is a huge cost difference between laser and exercise therapy.” (5)* *“A patient who approaches a government clinic, when sees that the doctor’s fee is less than a half cent, they visit another six specialists the same day. We are a tertiary center; we must all have referral letters; none of the patients have referral letters; a small percentage of them indicate to visit specialists; the rest are patients who, according to the algorithm of the Ministry of Health, should be handled by a general practitioner in an urban or rural health center.” (10)*	1, 4, 5, 6, 7, 8, 9, 10, 12, 13, 14, 15
	Legal challenges	*“Legal concerns and complaints are raised because medical practitioners do not want to miss a particular case. Otherwise, many health issues have specific algorithms, and professors are aware of these algorithms.” (3)* *“The examination of the patient is not documented. Therefore, it cannot be defended at the court, and when the patient complains, some issues can be caused for the medical practitioners, even if it is proven that they have not done anything wrong.” (3)* *“If patients ask for an MRI prescription and you don’t provide it, they will insult and threaten you, and you will inevitably prescribe it because the government will provide the doctor’s security.” (10)*	3, 7, 9, 10, 12
	Lack of effective supervision	*“There is no supervision on medical services.” (4)* *“Our subspecialized centers are tertiary centers, and the patient approaches with an MRI, so we have no role so far.” (6)*	4, 6

**Table 4:** Main facilitators in reducing low-value care in the management of low back pain in Iran

Main themes	Sub-themes	Quotes	Participant ID
**Individual provider characteristics**	Motivating the patients	*“The service providers can motivate patients by diversifying the suggested treatments.” (1)*	1,2, 4,9,15
	Compliance with ethical considerations	*“If a patient refers to me with low back pain from two days ago, I do not give the patient any appointments and suggest he be visited by other healthcare professionals. I do not prescribe painkillers and injections, but I am saying that it’s not in my carrier, and I cannot do anything about your condition.” (19)* *“In my opinion, depending on the medical practitioners’ values, sometimes money may be important to a medical practitioner, and sometimes morals.” (14)*	4, 14, 18,19
	Having a holistic view	*“In the physical medicine and rehabilitation specialty, we try to proceed based on history and physical examination to reduce the possibility of using low-value care interventions.” (7)*	4,8,7,19
	Adherence to clinical guidelines	*“In my opinion, the most important parameters to not prescribing low-value care interventions are the medical practitioners’ patience, clinical examination, and knowing the red flags.” (5)* *“Of course, in training clinics with residents, we try to follow the guidelines.” (7)* *“We all have to accept that we have to follow the guidelines in order to perform fewer surgical procedures and reduce surgical complications. The cost imposed on the health system and patients’ needs to be reduced.” (11)* *“Research has shown that in people who are healthy and have never experienced low-back pain, 80% have disc herniation signs in an MRI. For this reason, we try to evaluate the patient first, and if their condition does not improve with manual therapy and the symptoms become severe, we prescribe MRI; for example, in cases with bladder incontinence.” (14)*	1,5, 7, 9, 11, 12, 13, 14, 15, 17
	Developing an appropriate provider-patient relationship	*“If the doctors give the patient the proper awareness and sufficient information about exercise therapy and high-value care, in my experience, patients cooperate with me and do not need to visit me every day. This situation also reduces the economic burden on the health system and the patient. It also reduces the number of operations without indications.” (1)* *“According to physiotherapists’ idea, when medical doctors at the first stage recommend exercise, patients’ compliance increases significantly.” (11)* *“If the patient is convinced that there is no need to take quick and expensive measures at that stage, they can trust their therapist to continue their treatment.” (18)* *“The patients who have visited their doctor several times and are satisfied with their treatment accept everything we say and trust their doctor.” (9)* *“It is important to make trust between medical practitioners and patients.” (11)*	1, 4, 6,9 11,15, 18
	Considering an individualized approach	*“Even though the epidural injections that are performed have shown appropriate evidence, the case selection must be accurate; for example, we cannot prescribe epidural injections to all patients with low back pain. This treatment can indeed be effective, but this may be solved with medication, a course of exercise therapy, and a series of other less expensive management, and there may be no need to quickly prescribe the epidural injection.” (5)*	1,5,15, 18, 19
	Clarifying the duties of each professional	*“Everyone should intervene only in their area of expertise.” (12)*	1,12
	Supervising by peers	*“In the public sector, we share and discuss the number of surgical operations performed and their quality, which helps us monitor ourselves.” (10)*	1,10
**Individual patient**	Improving the cultural status of patients	*“The first thing that can be done in this field is to collateralized patients.” (2)* *“Culturalization is very important, and patients should be educated that not every case of low back pain needs an MRI to find a diagnosis.” (9)*	2, 3, 4, 7, 9, 11, 15,17,20
	Enhancing the awareness of patients	*“Patient education goes back to a layer before medical practitioners: the health management group, the hospital management group, the nursing group, and the education groups of the Ministry of Science.” (15)*	10, 15,20
	Increasing patients’ adherence to prescribed interventions	*“There should be an inhibitory law for the patient’s non-compliance with the provided treatment, not only for medical practitioners.” (10)*	3,10,20
**Social context**	Strengthening team-working	*“The teamwork experience in the public sector was very pleasant for all of us.” (1)* *“In some areas, surgeons have a good working relationship with the rehabilitation team, and if the rehabilitation treatments fail in the first stage, they direct the patients to a surgical operation.” (2)* *“Teamwork and communication between specialists should be expanded. For example, I have been invited to give a speech with neurosurgery residents about the rehabilitation aspect of the treatment of diseases.” (7)* *“They should set some restrictions before undertaking an operation or an MRI, which means that a multidisciplinary committee of several doctors and psychiatrists should be formed before that. For a patient who is undergoing surgery, sometimes the pain can be psychosomatic—that is, the patient undergoes surgery, and it gets worse. So, for any kind of treatment, the doctor alone should not be the decision-maker for the patient.” (11)*	1,2, 4, 7,8 11, 13, 14, 18
	Improving the cultural status of public	*“Education and culture at the level of the whole society, not only doctors and clinics, will significantly improve the use of LVC services.” (7)*	1,4, 5, 7, 9,17,20
**Organizational context**	Improving the medical educational system	*“All medical practitioners and even laypeople should be educated in order to ensure that extra care services are not imposed on the patient. Sometimes patients have a fear of being paralyzed, but this does not happen if the patients are aware.” (11)*	11
	Creating effective continuing training courses	*“There are some brochures that have been prepared to teach patients about food restrictions and effective exercises that give them reassurance.” (9)*	9
	Establishing a desirable payment mechanism	*“Everyone should be paid and supported financially based on their expertise and skills.” (1)* *“The value of the services we provide for patients and the time we spend for them should be calculated fairly. The income from Laser therapy and other therapeutic devices used in physiotherapy should not be more than a time-consuming thorough physical examination.” (12)*	1,4,10, 12,20
	Considering limitations in the use of imaging services	*“When a patient’s costs are covered by insurance and they do not need to bear any costs, and also services are available, when the patient knows that the MRI will be done the next day at the latest, they do not undertake conservative treatment and prefer to know the diagnosis sooner, or if surgery is to be done for them, to have it done sooner.” (8)*	8
	Incorporating high-value care in benefit packages	*“The insurance organizations should invest in the treatments recommended by the scientific guidelines—the documented base care—and set tariffs based on that, and not cover the services that are in the low-value care category.” (1)*	1, 12,18
	Improving referral system	*“If we have a proper referral system where the patient passes through different paths and finally can reach the surgeon, the economic burden on the health system and the patient will definitely be reduced.” (8)* *“The patient should not have direct access to specialists and subspecialists. They should first be visited by a general practitioner who visits general patients, and then, based on the history and physical examination, they should decide which specialist the patients should be referred to and then follow up to see if the conservative treatment given by the rehabilitation specialists has been implemented by the patient, and was the treatment effective?” (14)*	1,4, 5, 7, 8,10, 11, 12, 13, 14
	Clarifying interdisciplinary boundaries	*“We should have a guideline, or an algorithm based on common diseases for GPs so that they know up to what stage of the disease they can treat patients themselves when they should refer patients, and to whom? Everyone should intervene only in their area of expertise.” (5)*	1,5
	Effective management of available resources	*“In contrast to a private hospital, in the public sector, we do not have over management, and most patients do not undergo surgical operations.” (3)* *“Based on the economic conditions, resource management should be performed.” (7)*	3, 7,10
	Moving towards effective supervision and monitoring	*“Another measure that can be taken to help in this case and lead a person to make the right decision is supervision.” (1)*	1,4 9
	Promoting electronic information systems	*“With proper supervision and an electronic record, excessive requests and shortened visit times can be prevented. On the other hand, medical practitioners’ rights will not be disregarded.” (1)*	1
	Considering rationing methods	*“The health system should not support the patient’s request in all cases.” (1)* *“There should be some limitations on the number of requested medical services by medical practitioners or medical imaging officials, and there should be a long process to get an appointment to discourage patients.” (4)*	1, 4
	Strengthening ethical considerations of providers	*“We cannot claim that we work entirely based on morals. Money is needed, but not at any price. Therapists should put themselves in patients’ places. But the disproportion between costs and external expenses is influential in the patient’s treatment process.” (14)*	14
**The economic and political context**	Raising the medical tariffs	*“If doctors’ fees are really enhanced, the referral issue will be solved. If my visit fee as a* ***sub-specialist*** *is so expensive that the patient only visits me for specialist issues, then the problem will be solved.” (4)* *“When the tariffs are real, the demand decreases.” (10)* *“Tariffs should function as an automatic mechanism and primary motivation for deferring more complex specialized or subspecialized unnecessary services and minimizing low-value cares.” (5)*	1, 4, 5,10,20
	Creating motivation for providers	*“The medical practitioner should be motivated so that they feel that it’s cost-beneficial for them; not that they start doing freelance work after seven years of general medicine to support their family.” (13)* *“High-value cares such as lifestyle modification are often time-consuming for providers, they need to know that time is taken to count in term of financial reward as other interventions do.” (5)*	13,5,8
	Building a political advocacy strategy	*“As long as the people who have power and make laws do not want, this issue will not be solved.” (10)*	10
	Establishing binding laws	*“The requirement for its effectiveness is that it should be defined at the level of the Ministry of Home Affairs; something should be written, it needs to become a law, and decisions are made based on it with no bias.” (1)* *“Patient education should have executive context and support. And if they act against that, fines should be considered so that they cannot, for example, simultaneously make appointments with several specialists.” (10)*	1, 10
	Increasing the awareness of policy- and decision-makers	*“Even health policymakers should pay attention to these points.” (7)*	7,11
	Improving accountability and responsibility	*“As a surgeon, I am aware of the complications of spine surgery, and because it brings with it legislation for me, I refuse to perform it without an indication. So, if anyone is fully responsible for writing prescriptions, they will be less willing to do it.” (8)*	1, 7, 8, 10

### Main barriers in reducing low value care

The main barriers to reducing LVC interventions in the management of low back pain, according to the interviewees’ point of view, have been summarized in five related domains, including: (1) individual provider characteristics; (2) individual patient characteristics; (3) social context; (4) organizational context; and (5) economic and political context. We generated 35 subthemes from the data coded in these domains.

### Individual provider characteristics

Regarding characteristics related to the individual provider, non-adherence to clinical guidelines among doctors, physiotherapists, and even other healthcare staff and unawareness of providers regarding new techniques were recognized as barriers to the de-implementation of LVC for LBP.

*“Despite the progress in physiotherapy, physicians do not have proper knowledge about it. Many of our medical practitioners’ knowledge about physiotherapy is in the form of articles*, and since surgeons specialize in surgery, they often recommend surgical solutions.*” (Physiotherapist 14, 40 y/o, female)*.

Moreover, the lack of trust among involved professionals has also been mentioned by physiotherapist 14 as a barrier. Patient’s sense of ownership and tunnel vision are other factors result in self-centered decisions.

*“One thing that happens in our specialties is tunnel vision. Depending on their areas of expertise and the cases they typically visit, each doctor has a unique perspective on a patient. Low back pain may be more inflammatory for rheumatologists, or the mechanical aspect may be important for us, and we visit these patients more frequently.” (Physiatrist 5, 33 y/o, female)*.

Overtreatment can occur when practitioners fear missed diagnoses, and want to ensure their patient’s condition, especially when they are referring patients from distant cities (defensive medicine).

*“When a patient comes to me, I cannot refer them to another healthcare professional without providing any medical service due to the fear of missed diagnoses, which can lead to overtreatment.” (Physiatrist 5, 33 y/o, female)*.

The lack of a comprehensive physical examination was mentioned by many medical practitioners as a barrier. In a patient presenting with LBP, a thorough history and physical examination is essential to delineate the diagnostic and therapeutic plan.

*“… For example, if we perform the SLR and reverse SLR tests, we can to some extent understand whether LBP is associated with discopathy or radiculopathy. Based on the history and examination, some patients may have inflammatory spondyloarthropathies. We can order lab tests initially, and then if the pain doesn’t improve with conservative management, imaging and other costly services may be required.” (Rheumatologist 9, 33 y/o, female)*.

Time was another barrier mentioned by many participants. In Iran, there are regulations regarding the minimum visit time for specialists, although they are not usually followed.

*“If I want to spend five minutes explaining to each patient, I would spend six hours explaining to patients, and I don’t have this time in the clinic.” (Neurosurgeon 10, 36 y/o, male)*.

A lack of patient-centered management and the absence of specific treatment for each patient would cause patient insecurity about their care and time allocation. In some cases, the treatment plan is designed based on the condition of a certain group of patients. Many doctors are not thoroughly familiar with physiotherapy devices and request the same prescription for all patients (Physiotherapist 15, 32 y/o, female).

The use of corticosteroid injections without indication has increased these days, especially in rehabilitation settings and pain clinics. This approach is not necessarily due to a lack of physician knowledge about LBP management, but it may result from the perception of conservative therapy as a diagnostic failure by some patients.

*“The cost of corticosteroid injections is much higher than physiotherapy treatments. So, doctors prefer to treat their patients with a single injection session rather than long-term conservative treatments.” (Physiotherapist 1, 46 y/o, male)*.

### Individual patient characteristics

The most frequently mentioned barriers by the interviewees in the category of individual patient characteristics were lack of awareness, cultural difficulties, demanding non-invasive interventions, inappropriate patient cooperation with providers, willingness to get better quickly, and demand for receiving technology-based interventions. In this category, half of the interviewees mentioned distrust as the main barrier. One of the consequences of distrust among patients is the request for special medical services. Patients request MRI scans even in unnecessary situations due to a lack of trust in their doctors, and in some cases, doctors lack the required to convince their patients in this regard (Physiatrist 7, 43 y/o, Male).

Besides, sometimes patients visit doctors to request certain medical services. It happens when patients lack trust in their doctors. Many doctors aim to avoid patients’ dissatisfaction, as it may cause some issues for them. Therefore, they comply with patients’ requests (Orthopedic Surgeon 3, 30 y/o, Male). One likely reason for this issue is when patients receive information from unreliable sources.

*“Social media and misguided advertisements have reduced patient and doctor trust.” (Rheumatologist 9, 33 Y/O F)*.

Obtaining misleading or false health information from patients through the internet or from ordinary people may make patients distrustful of medical service providers. It is worth mentioning that malpractice can also cause distrust among patients. As stated by one of the neurosurgeons:

*“If patients experience malpractice, they lose their trust in medical professionals.” (Neurosurgeon 10, 36 y/o, Male)*.

Another interconnected point related to this issue is the lack of patient awareness about different medical specialties and their responsibilities. Medical specialty selection can be confusing and can affect the medical system’s performance. For example, one physiatrist noted that in the city where she works, patients are not familiar with her specialty, they are unaware of why they should see a physiatrist (Physiatrist 5, 33 y/o, Female).

Some patients do not adhere to their prescribed interventions, causing further issues for themselves and also leading to medical practitioners making mistakes.

*“Sometimes, patients visit several medical practitioners to get the desired prescription, which can lead to subsequent practitioners making mistakes.” (Physiatrist 11, 48 y/o, female).*

Therapeutic exercise, as a modality in the management of LBP, has low compliance among patients. This non-adherence or unwillingness to exercise among some patients can direct treatment toward unnecessary LVC treatment and diagnosis methods. At times, patients may lack the patience and motivation to undergo these non-invasive treatments for extended periods.

*“If we prescribe some exercises for six months, the patients do not like to do them (… are reluctant to follow them?)” (Physiotherapist 15, 32 Y/O F).*

In addition, patients do not cooperate with their healthcare providers in some cases: “Patients do not cooperate with medical practitioners” (Physiatrist 11, 48 Y/O F). This can be for several reasons, but it makes the situation more challenging for healthcare providers, and it may even influence their decision-making process. The interview responses regarding individual patient characteristics also pointed to cultural challenges as a factor leading to LVC for just under half of the participants:

*“In the sports medicine center where we worked, doing exercises was not culturally accepted as a treatment, and medications and injections were accepted as treatments” (Physiotherapist 1, 46 Y/O M).*

For example, some individuals believe that certain types of innovative technologies are superior and significantly impact the entire treatment and diagnosis process. Therefore, culture can play a significant role in promoting LVC.

Closely related to cultural challenges are the demands for technology-based interventions and the willingness to recover quickly. Two interviewees complained that some patients requested to receive technology-based interventions, which fall under the category of the barriers in the individual patient characteristics:

*“The more physiotherapy devices are used and the longer they are used, the more satisfied patients become.” (Physiotherapist 15, 32 Y/O F).*

Despite the beneficial effects of technology-based interventions, sometimes, the overuse or unnecessary use of these interventions’ conflicts with the de-implementation of LVC. Another barrier included was the patients’ willingness to recover quickly. Patients can become impatient sometimes. As explained by one of the participants, the patient may need non-surgical and long-term treatments, which require the cooperation of the patient to visit regularly, but they may prefer the final option, which is a surgical operation (Physiatrist 11, 48 Y/O F).

Demanding non-invasive interventions was only pointed out by one neurosurgeon as a patient-related barrier:

*“Patients’ desire for minimally invasive procedures without anesthesia, without incisions, even though without any clear indication.” (Neurosurgeon 8, 35 Y/O, M)”.*

### Social context

Identified barriers related to the social context included a lack of teamwork, inappropriate professional development, lobbying by some high-power professional networks, and the low socioeconomic status of patients. Half of the interviewees, including physiatrists, rheumatologists, physiotherapists, and orthopedic surgeons, noted that teamwork is not acceptable in our country, and many specialists prefer to manage their cases alone without referring them to other related specialties. Teamwork is not developed in our universities.

*“Teamwork is very weak in our country. It is mainly because, with the little knowledge that we gain, we tend to believe that we are experts in the field and can manage it ourselves, which prevents teamwork. We tend to comment on everything, and these interferences are largely a part of our sociology.” (Physiatrist 13, 40 Y/O, F)*.

The disproportionate popularity of certain disciplines among general population and even health care providers was another barrier mentioned by interviewees. Short medical student internship durations in some wards, the lack of physical medicine and rehabilitation wards in many hospitals, and the voluntary selection of some wards for internship in certain medical education programs may result in practitioners lacking sufficient information to refer a patient to the relevant specialties when they present with LBP.

*“An intern spends only fifteen days in the neurosurgery ward with a heavy workload, and quality training is not possible during this limited timeframe.” (Neurosurgeon 17, 33 Y/O, M)*.

Moreover, lobbying by a group of dominant specialties at the Ministry of Health level and the low socioeconomic status of patients, especially in the public sector, were mentioned as important barriers to the desirable management of LBP. Many interviewees pointed out that some patients insist on receiving certain services and may even threaten the doctor’s life if they are not provided with the requested services.

*“In recent years, when scheduling an appointment to see me has become more difficult, it is uncommon to see a patient with low back pain as the first line of treatment. As a result, the patient who come to me have already invested money and time in imaging and have made significant effort to visit other specialties. Because these patients are typically of low socioeconomic status, they expect both diagnosis and treatment to be completed during their initial visit.” (Physiatrist 7, 43 Y/O, M)*.

### Organizational context

Overall, nine main organizational barriers to reducing LVC interventions in the management of LBP were discussed in interviews. Some physicians and physiotherapists mentioned that sports medicine specialists and physiotherapists are not available in most health centers. Inadequate rehabilitation centers, on the one hand, an excessive number of imaging centers, on the other hand, pose significant organizational barriers for prescribing X-rays for non-specific LBP in the first visit.

The lack of a comprehensive referral system was one of the most commonly reported barriers. According to a physiatrist, even if our doctors know the correct principles of referral and management of LBP, due to this vicious economic, social, and cultural cycle, sometimes they have to initially take measures that logically are not carried out in the first place, like requesting imaging and laser therapy.

*“The referral system is not appropriate. Many patients with low back pain can visit surgeons or other specialists as the first step in their treatment process. So, at the first level, the patient should visit a GP, and they will be referred to a surgeon if they have red flags, or if physiotherapy intervention is needed, they should be referred to a physiotherapist.” (Physiatrist 12, 31 Y/O, M)*.

Lack of insurance coverage was among the most key factors in the organizational context, imposing a barrier. Most participants expressed their disappointment with insurance companies, as they cover only a limited number of infrequently used interventions. For example, it does not cover laser therapy, shock wave therapy, therapeutic massage, or exercise all of which are beneficial parts of the management parts of many patients with LBP. Whereas surgeries have much better insurance coverage. In addition to not covering many important interventions by the main health insurance companies in Iran, another issue that most health practitioners are faced with the insurance system is the delay in payments for six months to a year.

*“Unfortunately, insurance coverage for the treatment of low back pain is not favorable for either the therapist or the patient because a limited maximum of treatment cost coverage for the patients are offered. For example, there may be patients who need ten or more physiotherapy sessions per season, and the insurance companies do not cover it unless they have specific insurance plans.” (Physiotherapist 18, 36 Y/O, F)*.

Some physiatrists and physiotherapists have stated that they evaluate patients by thorough physical examinations and spend even more than half an hour teaching them therapeutic exercises and lifestyle modification tips, while there is no defined tariff for these services in the health system, and they are paid the same as their colleagues who spend much less time.

*“I think the main problem is time, and then for us, physiotherapists or surgeons, there is no fee to be charged for these high-value care services to spend time on and teach exercises and correct patients’ lifestyles.” (Physiatrist 5, 33 Y/O, F)*.

Another organizational barrier, mentioned by participants in distinct aspects, is conflicts of interest between different practitioners that inhibit referring their patients to other specialists. Moreover, there are mediators known as “medical tour leaders” who schedule appointments and accompany health tourists. They usually do not have certified medical knowledge and may interfere with the logical diagnostic and therapeutic approach for the sake of profit. (Physiatrist 7, 43 Y/O, M). Traces of conflicts of interest may be seen in even commonly used interventions.

*“The mafia power in the food and drug industries is under the control of those who import minimally invasive surgery equipment, and they try to increase the acceptance of minimally invasive surgery among patients and doctors.” (Neurosurgeon 18, 35 Y/O, M)*.

### Economic and political context

The economic and political context was presented in three main categories, including unrealistic tariffs, legal challenges, a lack of an effective supervision system, and limitations on prescribing medical services, which were stated by the majority of the participants as barriers.

Unrealistic medical tariffs are not limited to visits. For example, there is a huge cost difference between laser therapy and manual therapy. There is no established tariff for exercise therapy, which has a high level of evidence in treating low back pain.

*“Doctor’s fees, and medical services are at low costs. If my fee as a specialist is so high that patients can only visit me for special issues, then the referral system will be fixed.” (Rheumatologist 4, 57 Y/O, F)*.

Our professors are aware of medical guidelines and treatment algorithms, but since the physical examination cannot be documented as detailed as imaging; most practitioners request low-value interventions to not miss anything and can defend themselves in court when a patient sues them.

*“Legal concerns and complaints are raised because medical practitioners do not want to miss a particular case. Otherwise, many health issues have specific algorithms, and professors are aware of these algorithms.” (Orthopedics 3, 30 Y/O, M)*.

Main facilitators (solutions) in reducing LVC interventions in the management of LBP have been described in Supplementary file.

## Discussion

In this qualitative study, we discussed facilitators and barriers to reducing LVC for the management of LBP. These discussions were based on the perspectives of five groups of related specialists, including orthopedics, physiatrists, rheumatologists, neurosurgeons, and physiotherapists. It is important to understand each practitioner’s perception of their role within the healthcare system. The interviewees specified an extensive list of commonly used diagnostic tests and therapeutic procedures for LBP such as MRI and electrodiagnostic tests, analgesics, prolonged bed rest, some physical agent modalities, spinal injections, and surgical interventions that their perceived cost or risk of harm outweighed the expected benefits. This implies that, from the interviewees’ point of view, there is an abundance of LVC services being delivered for LBP. Although identifying these services is not enough to curb them [36], it is necessary to know them [36]. Due to the heterogeneity of etiologies and presentation of LBP, different diagnostic approaches and a wide range of therapeutic modalities and procedures have been proposed and are being used in daily practice. Clinical guidelines are developed to alleviate inappropriate variability in clinical practice. Integrating evidence and clinical experience with patient preferences is necessary for achieving high-quality care [37]. However, a clinical practice guideline alone does not guarantee the implementation of its evidence-based recommendations and the de-implementation of LVC. Therefore, it is logical to identify barriers and facilitators of implementation and de-implementation, which are challenging multifactorial processes. Although implementation and de-implementation seem to be two sides of the same coin, research findings on one are not necessarily transferable to the other [38].

In the present study, the interviews identified a number of barriers to reducing LVC in the management of LBP. Most participants stated individual provider characteristics as the important challenge in the management of LBP. Overall, unrealistic tariffs, followed by provider-induced demand, patient’s distrust, insufficient time allocation, lack of insurance coverage, lack of a comprehensive referral system, lack of teamworking, cultural challenges, lack of awareness, and defensive medicine were the ten most commonly cited barriers by the participants. Individual provider characteristics constitute a major cluster of barriers for reducing LVC [39]. Knowing these factors is crucial to engage practitioners in the de-implementation process. The study participants outlined practitioners’ preferences and financial motives as the determinants leading to provider-induced demand for certain health services. Practice routines and habits of clinicians cannot be easily modified, and clinicians’ resistance to change has been identified as a common barrier to reducing LVC [39]. Prevent bypassing referral system is one of the biggest challenges commonly reported in the literature. First, most practitioners like to follow the patient until the end of the treatment process. Second, Iranian health system is patient-centered. The patient himself or herself decides when and whom to meet and can easily access more than one specialty and subspecialty at the same time without any limiting regulations. While the patient should first see a GP, Iran’s health system does not fully support this [40]. The country’s week infrastructure for electronic data recording and the need to visit the doctor again is another barrier in term of organizational context that has mentioned by our participants. As Tabrizi et al. also stated that the primary health care information system needs to be transformed to the electronic system with personalized online health profile [41].

The interviews also identified 31 sub-themes as the main facilitators for reducing LVC in LBP management. However, the individual provider characteristics were the most frequently cited. Adherence to clinical guidelines, improving the referral system, improving the cultural status of patients, strengthening team-working, developing an appropriate provider-patient relationship, improving the cultural status of the public, motivating the patients, considering an individualized approach, establishing a desirable payment mechanism, and raising the medical tariffs were the ten most commonly represented facilitators in the interviews. As can be seen, some factors have been expressed in diverse ways, both as a barrier and a facilitator. For example, the lack of a suitable referral system is mentioned as a barrier and improving the referral system as a facilitator. The interviewees suggested an individualized approach based on each patient’s needs and not as a part professional’s routine as a facilitator for reducing LVC in LBP.

Free of charge services and the availability of imaging services have been stated as the greatest strength of our health care system. In Contrast, according to the point of view of some interviewees, it may cause overtreatment and waste of resources. In tertiary centers, according to the algorithm of the Ministry of Health, all patients should have a referral letter. Patients do not only have referral letters from their family physicians, but they also visit many other specialties since the fee is less than a cent. When a patient’s costs are covered by insurance, and they do not need to bear any expenses, and when services are readily available, they do not undertake conservative treatment and prefer receive an earlier diagnosis [41].

The influence of economic incentives on the practice of healthcare professionals as the driver of LVC has been discussed [42, 43]. Consistent with the literature, the participants reported workload, time constraints, insufficient time for explaining the necessary points to patients, and ordering unnecessary para-clinical investigations like MRI scans to compensate for inadequate clinical assessment [42, 44–47] as individual provider-related determinants. The substantial number of patients in government and teaching hospital clinics would shorten the time for educating users regarding proper exercises, conservative treatment, and lifestyle modification in the management of LBP. Therefore, lack of sufficient time is a major reason for the difficulty of practitioners’ behavior change [48].

The concept of defensive medicine emerged more than 50 years ago, representing the practice of medicine primarily aimed at lowering the risk of malpractice litigation [49]. In a cross-sectional study on a group of GPs in Iran, the frequency of positive defensive medicine was 99.8%, which is the highest occurrence rate among the studied countries in the review done by Kakemam et al. [50, 51]. In one survey, fear of malpractice was the most frequent reason physicians ordered LVC [52]. In a scoping review of the studies on determinants for the use or de-implementation of LVC, professionals’ fear of malpractice was a commonly identified reason for providing LVC [53]. The proper malpractice insurance coverage, along with effective legislation to protect practitioners may help reduce their concerns about malpractice. Individual providers’ non-adherence to clinical guidelines was not a frequently cited barrier in our series. However, as a facilitator, the adherence to clinical guidelines was the most commonly noted factor. In a systematic review of 960 studies from more than 20 countries, at the healthcare professional level, lack of knowledge was the primary barrier to the implementation of clinical practice guidelines. At this level, education was the most commonly cited facilitator [54]. Unlike some studies, the participants did not point out provider characteristics such as age, gender, clinical experience, and personality.

Ongoing training courses, including medical education and programs for healthcare professionals, are crucial for keeping healthcare workers up-to-date and adaptable to changing healthcare needs [55]. These initiatives demonstrate a commitment to providing accessible and high-quality healthcare services for all patients. Initial or continuing healthcare professionals’ education regarding the proper management of low back pain has shown to be important and impressive, especially in the context of Iran’s evolving healthcare system. The Universal Health Coverage program focuses on continuous education, recruiting local health workers, and improving the capabilities of existing staff. The program ensures that GPs and other healthcare workers receive free education to promote their skills and knowledge. By analyzing and revising the education system, Iran aims to enhance the overall competence of healthcare professionals [56, 57].

The “Unrealistic tariffs” was the most frequently stated barrier by the participants. The visit-based, fee-for-service is the primary payment mechanism for private-sector physicians in Iran. The tariffs have not risen at the same pace as the recent high inflation rates. Low fees may incentivize practitioners to increase their activity and lead to providing unnecessary services [42]. Raising the medical tariffs was one of the most repeatedly mentioned facilitators for the de-implementation of LVC in LBP. It has also been argued that the fee-for‐service payment mechanism leads to more low‐value services [58]. Therefore, establishing a desirable payment mechanism based on the skills and expertise of healthcare professionals and considering the value of their time spent was recommended to reduce LVC services.

Distrust was the most frequently mentioned barrier in the individual patient characteristics category. Indeed, distrust generally occurs when patients assume substandard healthcare is provided for them [59]. Thus, building trust between the patient and clinician is important for effective communication and a facilitator for reducing LVC [60]. Effective communication, on the other hand, is needed to convince patients who ask for LVC services. In this study, social media and misguided advertisements were reported to be responsible for decreased trust in the physician–patient relationship. Apart from the influence of trust on providing LVC, patients with higher trust in their clinicians report better health outcomes [61]. Therefore, it is essential to identify the causes of distrust and adopt approaches to build and maintain patient-clinician trust.

Iran is a multicultural community with many ethno-linguistic groups [62]. Previous studies have shown variations in the healthcare choice behavior of different cultural groups [63]. Our participants pointed out circumstances in which patients do not accept prescriptions and advice like painkillers, therapeutic exercises, and short course rest sufficient for their problem and seek other remedies such as injections or imaging studies like MRI. Participants also repeatedly cited the lack of medical knowledge of patients as a main barrier to reducing LVC in the management of LBP. For instance, this issue appeared as the unawareness of patients about some medical fields like rehabilitation. On the other hand, expert patients with superficial knowledge may ask for services considered LVC by the practitioners [64]. These patients usually request certain services, especially those of higher technology.

Additionally, lack of teamwork was the most frequently stated barrier in the social context. Despite the unequivocal emphasis of medical literature on the importance of teamwork, controversies continue over the definition of healthcare professionals’ teamwork. To be more inclusive, different “inter-professional activities to provide safe and effective care” was considered the basis of a broad recent definition [58]. From the participant’s quotes is inferred that by teamwork they did not necessarily mean formally constituted teams, but they considered inter-professional cooperation activities like referral or consultation as teamwork. Inappropriate referrals were also discussed in the organizational context. Indeed, the lack of a comprehensive referral system was one of the most repeatedly stated barriers. In the absence of a formal referral mechanism based on family physicians, the patient-centered system allows unnecessary specialist visits, leading to probable low-value services. The sense of ownership of patients was stated as an individual provider characteristic. This attitude leads to practitioners’ tendency to follow patients to the end of treatment by themselves. McFubara [65] has discussed the ethical aspects of the concept of ownership of the patients emphasizing patients’ safety and protection as the prime concern of health professionals. Overall, as one participant stated, inappropriate referral system is the result of a diverse group of factors, including economic, cultural, and social determinants.

The role of GPs in reducing LVC interventions is notable in many health systems around the world. The Australian health system implanted an LBP program, by addressing low-value-care interventions in the primary care setting, aimed to reduce the amount of improper GP referrals for radiography. The outcome of this program showed reduced economic burden not only on the health system but also less patient’s exposure to health risks [66]. On the other hand, a literature review by Mousavi and colleagues compared the current management of LBP in Iran with practices recommended by recent evidence-based clinical practice guidelines, noted that there is no established patient referral system in our country. The majority of individuals experiencing acute or chronic LBP tend to bypass GPs and directly seek consultation with orthopedic surgeons, neurosurgeons, or rheumatologists. This highlights a trend where GPs play a relatively unimportant role in the initial management of LBP cases in Iran [67].

Another important aspect in the management of LBP which was not thoroughly mentioned by the interviewees is fear-avoidance beliefs not only in the patients but also among the healthcare professionals. Fear-avoidance Beliefs refer to the fear-based avoidance of movements or activities and have been proposed as a key mechanism in the development of chronic LBP issues [68]. These beliefs are associated with the severity and chronicity of the LBP as well as the time to return to work, and the outcome of rehabilitation programs [69, 70]. The healthcare professionals’ “fear” of delivering clear information regarding activities, pain, and return to work may be linked to “avoidance” [71]. Several studies evaluated the effect of healthcare professional beliefs on patients’ outcomes. They showed that GPs and physiotherapists with a treatment approach featuring frequent recommendations for bed rest and analgesics as required had patients with considerably higher disability at follow-up than practitioners who advise self-care measures [72].

### Limitations

Despite the efforts of the research team, this study also faces several limitations. First, only service providers were interviewed in the current study, which could result in this study lacking the perspectives of policymakers and service recipients. Therefore, it is necessary to explore the perspectives of health policymakers and LBP patients in relation to the most important barriers and facilitators of prescribing low-value services in future studies. Second, the geographic setting of the study was one of the provinces of Iran (Fars), which may restrict the generalizability of the findings expressed by participants. Third, the duration of some interviews in our study was shorter than others, and a subset of interviews was conducted via telephone, which may raise concerns regarding the quality of the interviews. These variations were influenced by multiple factors, including time constraints, internet connection limitations, participant preferences, and differences in the pace of speech.

## Conclusion

In conclusion, this qualitative study has pointed out a great number of barriers and facilitators that shape the provision of LVC in the management of LBP in Iran. Drawing insights from diverse healthcare specialists, we have identified a range of factors contributing to the persistence of LVC, including individual provider characteristics, economic incentives, patient distrust, time constraints, and inadequate teamwork. Conversely, potential avenues for reducing LVC lie in strategies such as adhering to clinical guidelines, improving the referral system, building patient-provider trust, and establishing suitable payment mechanisms. Therefore, it is essential for relevant stakeholders to consider these findings in order to de-implement LVC interventions in the process of LBP management.

### Supplementary Information

**Additional file 1.**

## Data Availability

The datasets used and/or analyzed during the current study are available from the corresponding author on reasonable request.

## Abbreviations

HVC: High-value care
LVC: Low-value care
LBP: Low back pain
MRI: Magnetic Resonance Imaging
