# Supplementary material for: Barriers and facilitators to reducing low-value care for the management of low back pain in Iran: a qualitative multi-professional study
Source: BMC Public Health. 2024 Jan 17;24:204. doi: 10.1186/s12889-023-17597-1 (PMC10792884; doi:10.1186/s12889-023-17597-1)
Supplement: Supplementary file 1 — Additional file 1. [file 12889_2023_17597_MOESM1_ESM.docx]

**Supplementary file:** Continuation of the results section

**Main facilitators in reducing low value care**

Main facilitators (solutions) in reducing LVC interventions in the management of LBP according to the interviewees have been summarized in five related domains, including: 1) individual provider characteristics; 2) individual patient characteristics; 3) social context; 4) organizational context; and 5) economic and political context. We identified 32 subthemes from the data coded in these domains.

**Individual provider characteristics**

Provider-related facilitating factors were motivating the patients, compliance with ethical considerations, having a holistic view, adherence to clinical guidelines, developing an appropriate provider-patient relationship, considering a patient-centered approach, clarifying the duties of each professional, and supervising peers. Adherence to clinical guidelines is an important indicator for effective and successful medical decision-making. A substantial proportion of participants considered adhering to the clinical guidelines as an essential element of reducing LVC. According to one of the participants commenting on the importance of clinical guideline adherence, we may miss some cases, but we will have fewer operations, surgical complications, and costs in return (Physiatrist 11, 48 Y/O, F).

Developing an appropriate relationship between patients and providers was considered an essential facilitator to reducing LVCs. Building a culture of trust was perceived as a necessary item for reducing LVC. This relationship guides patients toward appropriate treatment and increases the quality of the care provided.

*“If the doctors give the patient the proper awareness and sufficient information about exercise therapy and high-value care, in my experience, patients cooperate with me and do not need to visit me every day. This situation also reduces the economic burden on the health system and the patient. It also reduces the number of operations without indications” (Physiotherapist 1, 46 Y/O, M).*

A quarter of interviewees considered the individualized approach an effective strategy in this category:

*“Even though the epidural injections that are performed have shown appropriate evidence, the case selection must be accurate. For example, we cannot prescribe epidural injections to all patients with low back pain. This treatment can indeed be effective, but this may be solved with medication, a course of exercise therapy, and a series of other less expensive management strategies, and there may be no need to quickly prescribe the epidural injection” (Physiatrist 5, 33 Y/O, F).*

A few interviewees agreed that by motivating patients, we could facilitate reducing LVC in the management of LBP. One way to increase motivation in patients based on a physiotherapist’s opinion is by diversifying the available treatment options (Physiotherapist 1, 46 Y/O, M).

To reduce the possibility of using LVC interventions, some of the medical providers mentioned compliance with ethical considerations as a crucial factor. Ethical consideration is a core component of providing effective health care. Two physiotherapists and two rheumatologists commented on this sub-category; for instance, one physiotherapist noted:

*“In my opinion, depending on the medical practitioners' values, sometimes money may be important to a medical practitioner, and sometimes morals” (Physiotherapist 14, 40 Y/O, F).*

Under the category of individual provider characteristics, supervision by peers and clarifying the duties of each professional were also considered important by a few participants. It is important for all medical providers to get involved and provide treatments in just their field of expertise. However, sharing opinions and experiences can be an effective way to improve patient care. For example, a neurosurgeon explained that sharing and discussing some surgeries performed and their outcomes can help colleagues monitor each other (Neurosurgeon 10, 36 Y/O, M).

**Individual patient characteristics**

Almost half of the interviewees stated that improving the cultural status of patients is a crucial factor in the success of de-implementation. They believed it should be accomplished by approaches like enhancing public awareness and education. The cultural aspects of patient characteristics were discussed as an important determinant of using LVC in the management of LBP.

*“Modifying cultural misbeliefs is very important, and patients should learn that all cases of low back pain do not need an MRI to reach a diagnosis.” (Rheumatologist 9, 33 Y/O, F)*

In addition to the role of the provider in giving adequate information on treatment options, some participants mentioned the influence of media on patients' knowledge. The importance of educating patients about LVC and providing reliable information through the media was emphasized. Additionally, the practitioners stressed increasing patients’ adherence to prescribed interventions.

**Social context**

Under the social context category, strengthening teamwork was mentioned by the majority of interviewees. Based on their experience of teamwork and “especially the very favorable results in the public center,” they believed that multidisciplinary groups of different specialists should be formed, and the first doctor should not be the only decision-maker.

*“In some areas, surgeons have a good working relationship with the rehabilitation team, and if the rehabilitation treatments fail in the first stage, they refer the patient to a surgeon.” (Physiatrist 2, 35 Y/O, F)*

Moreover, improving the cultural status of society by focusing on public education was another important topic mentioned by rehabilitation practitioners in the social context.

*“Education and improving the culture at the level of the whole society, not just healthcare personnel, will significantly affect the use of LVC services.” (Physiatrist 7, 43 Y/O, M)*

**Organizational context**

In the organizational context category, fifty percent of interviewees cited the importance of improving the referral system. They criticized direct patient access to specialists and subspecialists. They believed that the most appropriate referral system is an early referral from GPs to other healthcare providers, when necessary, which can benefit patients and improve the quality of care. Many patients with LBP consult a surgeon and specialist at the first step. Patients need to refer to GPs at the first level. If they exhibit red flags, they should be referred to a surgeon, and if they need physiotherapy, they should be directed to a physiotherapist (Physiatrist 12, 31 Y/O M). According to a neurosurgeon, a referral system that guides patients through the appropriate pathway, ultimately leading them to a surgeon, can reduce the economic burden on both the healthcare system and the patient (Neurosurgeon 8, 35 Y/O, M).

Interviewees described improving the medical educational system, continuing education, and providing patients with relevant information as strategies to reduce LVCs:

*“All medical practitioners and even laypeople should be educated in order to ensure that extra care services are not imposed on the patient. Sometimes patients have a fear of being paralyzed, but this does not happen if the patients are aware” (Physiatrist 11, 48 Y/O, F).*

*“Some brochures can be prepared to teach patients about food restrictions and effective exercises” (Rheumatologist 9, 33 Y/O, F).*

Participants discussed many aspects of how the payment mechanism can affect LVC. They mentioned issues relating to medical tariffs supporting some LVC interventions to be used more frequently:

*“The value of the services we provide for patients and the time we spend for them should be calculated fairly. The income from laser therapy and other therapeutic devices used in physiotherapy should not exceed the reimbursement for a time-consuming thorough physical examination” (Physiatrist 12, 31 Y/O, M).*

In the same way, participants discussed the ways HVC could be incorporated into health insurance benefit packages and how to support them in the medical system:

*“Insurance organization plans often overlook chronicity and recurrence of the patients' condition. They should offer plans including long-term, high value care*” *(Physiotherapist 18, 36 Y/O, F)*

Clarifying interdisciplinary boundaries was stated as a determinant factor in reducing LVC:

*“We should have guidelines or algorithms for GPs so that they know up to what stage of the disease they can treat patients themselves, when they should refer patients, and to whom? Everyone should intervene only in their area of expertise “(Physiatrist 5, 33 Y/O, F).*

Among participants, a physiotherapist discussed the ethical aspects of using LVCs among providers. She recommended that therapists should put themselves in patients’ shoes and consider morals in patient care (Physiotherapist 14, 40 Y/O, F).

Some participants, also noted the importance of promoting electronic information systems, moving toward adequate supervision and monitoring, effective management of the available resources, and considering rationing strategies.

**Economic and political context**

Regarding the economic and political context, raising medical tariffs, creating motivation for providers, building a political advocacy strategy, establishing binding laws, increasing the awareness of policy- and decision-makers, and improving accountability and responsibility were identified as facilitators in reducing LVC in the management of LBP.

Participants commented that setting the right medical tariffs is a factor in balancing the load of patients’ visits and demands and decreasing unnecessary services. Indeed, the existence of rational tariffs for different medical services can increase the quality of health service delivery and minimize the overall cost of LVC accordingly.

*“If doctors' fees are enhanced, the referral issue will be solved. If my visit fee as a sub-specialist is so expensive that the patient only visits me for specialist issues, then the problem will be solved” (Rheumatologist 4, 57 Y/O, F).*

Additionally, healthcare providers should be motivated in terms of financial gains and satisfaction. As pointed out by one participant, for medical practitioners to be motivated to practice, they should feel that it is cost-beneficial for them to do so. (Physiatrist 13, 40 Y/O, F).

In terms of rules and regulations, two interviewees cited establishing binding laws as an effective action to be taken by policymakers.

*“Patient education should have an executive context and support. In cases where patients act against it, fines should be considered to prevent situations like making appointments with several specialists simultaneously” (Neurosurgeon 10, 36 Y/O, M).*

Increased awareness among policymakers was another strategy stated in this category. For example, given our current economic situation, it is necessary for us to do resource management (Physiatrist 7, 43 Y/O, M). At the same time, building a political advocacy strategy was said to play a necessary role in reducing LVC:

*“As long as the people who have power and make laws do not want it, this issue will not be solved” (Neurosurgeon 10, 36 Y/O, M).*

Being responsible for the type of service provided by healthcare providers can affect the treatment outcomes. As noted by some of the participants, improving accountability and responsibility helps deter substandard practices:

*“As a surgeon, I am aware of the complications of spine surgery, and due to legal concerns, I refuse to perform it without a clear medical indication. So, if anyone is fully responsible for their practice, they will be less willing to proceed with it” (Neurosurgeon 8, 35 Y/O, M).*
